# Subcapital Femoral Neck Fracture Despite Cement-Augmented Cephalomedullary Nail Fixation for an Osteoporotic Intertrochanteric Fracture: A Case Report and Position- and Sliding-Based Decision Guide

**DOI:** 10.3390/clinpract16010001

**Published:** 2025-12-22

**Authors:** Suguru Yokoo, Yukimasa Okada, Kyotaro Ohno, Takahiko Ichikawa, Chuji Terada, Keiya Yamana

**Affiliations:** 1 Department of Orthopaedic Surgery, Fukuyama City Hospital, Hiroshima 721-8511, Japan; 2 Department of Pathology, Fukuyama City Hospital, Hiroshima 721-8511, Japan

**Keywords:** cephalomedullary nail, bone cements, helical blade, femoral neck fractures, calcar-referenced TAD, varus deformity, telescoping, total hip arthroplasty

## Abstract

**Background/Objectives:** Cement augmentation of cephalomedullary head elements can improve the purchase of osteoporotic bone; however, it does not eliminate the need for accurate implant positioning or the preservation of sliding. We report the case of an 87-year-old woman who underwent intramedullary nailing with a cement-augmented helical blade for intertrochanteric fracture. **Methods:** This is a single-patient case report. Calibrated radiographic measurements—tip–apex distance (TAD), calcar-referenced TAD (CalTAD), neck–shaft angle (NSA), and telescoping—were obtained immediately postoperatively and at 4, 7, 12, and 15 months. CT was performed at postoperative week 1 and at failure, and MRI was performed for clinical deterioration. In addition, a targeted narrative review summarizes the evidence on the head-element position, sliding behavior, reduction alignment, and augmentation. **Results:** Immediate postoperative indices were within the accepted targets: TAD 22.6 mm, CalTAD 22.8 mm, NSA 134°, with the head element inferior on the anteroposterior view and central on the lateral view. Rehabilitation proceeded with full weight bearing as tolerated. Early telescoping was minimal (3.8–3.9 mm). Between 7 and 15 months, progressive varus with shortening of TAD/CalTAD and little additional telescoping was observed, radiographically consistent with relative proximal migration of the head–cement complex and a cleavage plane along the inferior cement mantle, culminating in a subcapital femoral neck fracture with the implant in situ. Emphasis should be placed on accurate implant positioning and preservation of sliding capacity, because cement augmentation alone may not prevent mechanical failure when the implant position or load transfer is suboptimal. **Conclusions:** Cement augmentation stiffens the interface and reduces micromotion but does not neutralize malposition-induced stresses. Accurate positioning, preservation of sliding, and timely conversion when sliding fails to progress are advisable; these findings are hypothesis-generating from a single case. We propose a position- and sliding-based decision guide to support clinical decision-making; its usefulness remains to be validated in larger studies.

## 1. Introduction

Trochanteric and subtrochanteric fractures in older adults are commonly treated using cephalomedullary nails or sliding hip screws [1,2,3]. Despite advances in design and perioperative care, fixation failure in severely osteoporotic bones remains a clinically important issue [4,5]. To improve purchase in weak cancellous bone, cement augmentation of the cephalic element has been adopted, and is associated with fewer “classic” failures, such as cut-out or varus collapse, in selected patients [6,7,8].

An uncommon but clinically significant complication after successful fixation is a femoral neck fracture with the implant still in situ. In a systematic review of 104 reported cases, the median incidence was 0.43% and the patients were typically elderly (mean age, 80 years). The review emphasized that the only consistently prevalent factors were (i) systemic/local osteoporosis and (ii) the presence of a cephalic implant traversing the neck or head. “Short” or high-positioned head elements can act as stress risers, but two-thirds of fractures still occurred despite subchondral placement; varus reduction/nonunion and screw breakage were additional contributors, and most events were atraumatic. The etiology of this condition is multifactorial [9].

Successful proximal femoral load transfer depends not only on the implant–bone interlock but also on the head element position and reduction quality. Established metrics, such as the Cleveland 9-zone position, tip–apex distance (TAD), and calcar-referenced TAD (CalTAD), capture these principles; malreduction in varus or a superior/anterior trajectory increases bending and shear across the neck and subchondral plate [10,11,12].

Cement augmentation stiffens the head–bone interface mainly by reducing micromotion with limited stress reduction, especially near the cement border [13]. Therefore, the anterior/superior trajectory or varus alignment still concentrates on bending/shear, and cannot be rescued by augmentation alone. Additionally, cement can create local stress-riser effects that relocate the peak stresses to adjacent trabeculae [14,15].

Herein, we report a case of a femoral neck fracture after intramedullary fixation using a cement-augmented helical blade. Despite the apparently robust purchase and initially acceptable positioning, the construct drifted into progressive varus with constrained telescoping, and neck failure occurred. This case was paired with a focused mini-review to reinforce the practical message that careful implant positioning and preservation of sliding capacity are critical, and to integrate position metrics (Cleveland zones, TAD/CalTAD) with pragmatic salvage options into a simple, decision-oriented framework for osteoporotic hip fracture care.

## 2. Materials and Methods

### 2.1. Ethics and Settings

This study adhered to the Declaration of Helsinki and was approved by the Institutional Review Board of Fukuyama City Hospital (approval no. 846; 29 November 2024). The requirement for informed consent was waived in accordance with institutional policy. This is a single-patient case report from a tertiary referral hospital.

### 2.2. Index Procedure and Rehabilitation

Closed reduction and antegrade cephalomedullary fixation were performed under fluoroscopic guidance using the TFN-ADVANCED™ Proximal Femoral Nailing System (TFNA; DePuy Synthes, West Chester, PA, USA). A helical blade was inserted, and cement augmentation was applied using the manufacturer’s system. Distal fixation involved the use of a single static interlocking screw. Postoperative care included next-day mobilization with weight-bearing as tolerated and pharmacologic thromboprophylaxis, according to the institutional protocol.

### 2.3. Imaging and Measurements

Standard anteroposterior (AP) hip and cross-table lateral radiographs were obtained during the routine follow-up. CT scans were acquired at postoperative week 1 to confirm cement distribution and the quality of reduction, at 12 months when mild pain and progressive deformity were first noted, and at failure to delineate the fracture pattern and the relationship between the fracture, cement mantle, and implant. To limit radiation exposure, additional cross-sectional imaging at shorter intervals was avoided. MRI (coronal T1; coronal T2 fat-suppressed/Dixon-water) was performed at the time of clinical exacerbation to detect subchondral low-signal bands suggestive of early osteonecrosis and to assess bone marrow edema or stress reaction related to progressive mechanical failure. The measurements were performed using the institutional PACS (SYNAPSE; FUJIFILM Corporation, Tokyo, Japan) by two independent readers calibrated to a known helical blade width (10.35 mm). Telescoping (sliding distance) was defined as the change in blade protrusion relative to the barrel between time points. The head position was classified using the Cleveland 9-zone grid [11]. TAD was measured according to Baumgaertner et al. [12] and CalTAD was calculated as described by Caruso et al. [10]. The neck–shaft angle (NSA) and qualitative telescoping (tip migration) were recorded. We reserve barrel-based telescoping for radiographic barrel sliding on AP views, and at the CT time points (12 and 15 months) we also list CT-based apparent blade protrusion as a descriptive index of lateral blade migration due to relative head–cement proximal migration, which is not directly comparable to telescoping.

### 2.4. Histopathology

During conversion arthroplasty, the femoral head was decalcified, paraffin-embedded, and stained with hematoxylin and eosin (H&E). Representative blocks were used to sample the subchondral plate, multiple marrow regions, and the adjacent cement tissues. Final magnifications are provided in the figure legends.

## 3. Case Presentation

### 3.1. Patient Information

**Figure 1:**
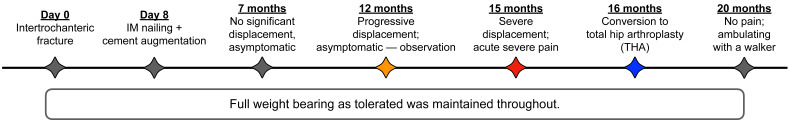
Clinical timeline of the case. The patient sustained an intertrochanteric fracture that was treated with cement-augmented intramedullary nailing (day 8). At 7 months, no radiographic displacement or pain was observed. At 12 months, progressive displacement was observed; however, the symptoms remained mild, and conservative observation continued. At 15 months, the patient experienced severe pain with radiographic progression, and MRI at that time did not reveal a T1 subchondral low-signal band. The patient underwent conversion to total hip arthroplasty 16 months later. Pathological examination revealed negative results for avascular necrosis (AVN). Full weight bearing was maintained throughout the study. Color-coded diamonds indicate clinical status: gray, routine follow-up events; orange, progressive displacement under observation; red, acute severe pain; blue, conversion to total hip arthroplasty (THA).

An 87-year-old woman with established osteoporosis (lumbar spine [L1] bone mineral density 0.665 g/cm^2^, T-score −2.6) sustained a low-energy fall at home. She was initially admitted to a referring hospital, where transient dehydration and her general condition were corrected. She was transferred to our center on post-injury day 7 and underwent surgery on day 8 (Figure 1). Baseline radiography confirmed a left intertrochanteric fracture of the proximal femur that required surgical fixation. Premorbid, the patient ambulated independently indoors using a cane.

### 3.2. Index Operation

**Figure 2:**
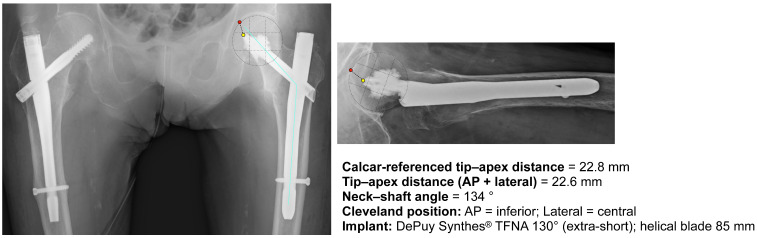
Immediate postoperative radiographs with position assessment (AP and lateral). Immediate postoperative AP (**left**) and lateral (**right**) radiographs with overlays for position assessment are shown. The Cleveland grid indicates that AP = inferior and lateral = central. The measured values were displayed in the measurement box. Implant: DePuy Synthes^®^ TFNA 130° (extra-short). The blue line indicates the axis used to calculate the neck–shaft angle, and the red and yellow dots indicate the points used to measure TAD.

Antegrade cephalomedullary nailing with a cement-augmented helical blade (DePuy Synthes^®^ TFNA 130°, extra-short; blade length, 85 mm) was performed on post-injury day 8. Immediate postoperative radiographs (AP and lateral) showed the head element in the inferior position on AP and central on lateral (Cleveland grid), with a CalTAD of 22.8 mm, conventional TAD of 22.6 mm, and NSA of 134° (measurement calibration is described in Section 2.3). This reduction was maintained (Figure 2).

### 3.3. Postoperative Course and Serial Imaging

Rehabilitation proceeded with full weight bearing as tolerated (WBAT). Functionally, at 4 months, the patient required a wheelchair but was able to stand; at 7 and 12 months, she ambulated with a walker; and at 15 months, worsening groin pain rendered her non-ambulatory.

**Figure 3:**
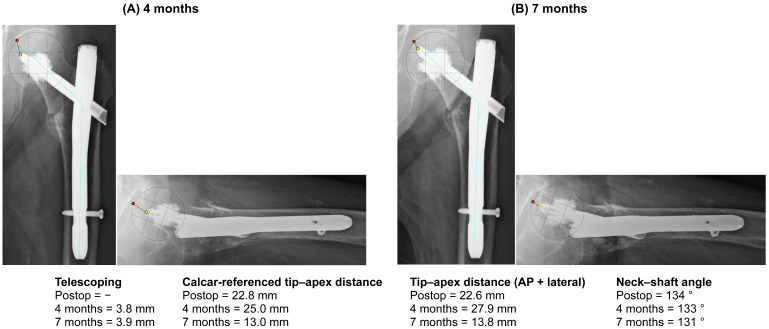
Follow-up radiographs (4 and 7 months) showing progressive varus. (**A**) 4 months radiographs. (**B**) 7 months radiographs. Telescoping measured relative to the barrel was 3.8 mm at 4 months and 3.9 mm at 7 months, and the apparent TAD/CalTAD shortening reflects en bloc blade-superior-mantle migration with inferior-mantle cleavage, rather than true barrel telescoping. The blue line indicates the axis used to calculate the neck–shaft angle, and the red and yellow dots indicate the points used to measure TAD.

Radiographically, at 4 months, the construct was largely stable, with minimal telescoping (3.8 mm) and a slight decrease in the NSA (133°). At 7 months, the patient remained asymptomatic; telescoping was essentially unchanged (3.9 mm), whereas the measured TAD and CalTAD showed apparent shortening (TAD 27.9 → 13.8 mm; CalTAD 25.0 → 13.0 mm) (Figure 3, Table 1). The combination of minimal telescoping with apparent shortening of tip–apex distances is most consistent with the relative proximal migration of the head–cement complex (i.e., apparent en bloc migration of the blade with the superior cement mantle and a cleavage along the inferior mantle) rather than simple controlled sliding; however, this interpretation is radiographic and inferential in nature. Progressive varus drift to a NSA of 131° was also observed.

**Figure 4:**
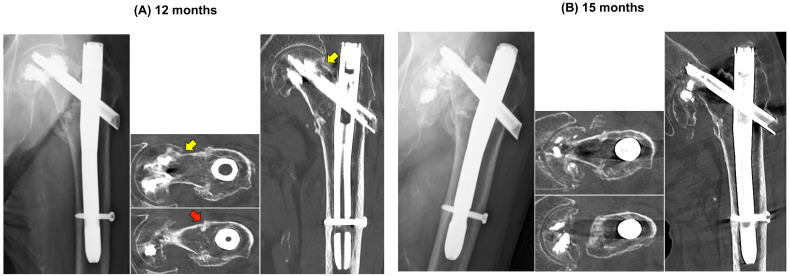
12- and 15-month imaging: progression to a subcapital femoral neck fracture. (**A**) 12 months: AP radiograph shows pronounced head–neck varus with en bloc blade–superior cement mantle migration and no interval barrel-based telescoping. Axial CT at 12 months demonstrates a linear superior subcapital sclerotic band (yellow arrow) and shows union across the intertrochanteric region (red arrow). (**B**) 15 months: AP radiograph and CT reconstructions—axial and coronal, bone window—confirm an ipsilateral subcapital femoral neck fracture with fragmentation; the intertrochanteric region remains united.

At 12 months, radiographs demonstrated a pronounced head–neck varus tilt relative to the shaft, with en bloc cephalad migration of the blade and superior cement mantle and no interval barrel-based telescoping, consistent with evolving subcapital failure rather than intertrochanteric collapse (Figure 4A). Given the modest activity-related discomfort and the patient’s preference to avoid further surgery, we adopted close clinical and radiographic surveillance with WBAT. At 15 months, the patient’s groin pain had acutely worsened without new trauma, and imaging confirmed an ipsilateral subcapital femoral neck fracture with marked fragmentation adjacent to the cement-augmented head (Figure 4B). Representative axial and coronal CT reconstructions at failure delineated the fracture geometry and spatial relationships between the residual head fragments, cement mantle, and implants. Because of extensive fragmentation and metal–cement artifacts, formal NSA/TAD measurements at the time of failure were not feasible.

### 3.4. MRI at Deterioration

**Figure 5:**
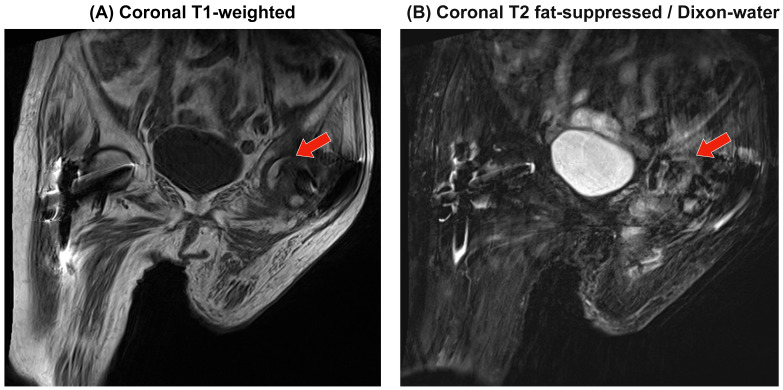
MRI of the hip: T1-weighted and T2 fat-suppressed (Dixon). Coronal T1-weighted (**A**) and coronal T2 fat-suppressed (Dixon water) (**B**) images of the affected hip are shown. No definite subchondral low-signal band was identified on T1; T2-FS demonstrated a heterogeneous marrow signal without focal collapse. The images were limited by motion artifacts and constrained hip extension. Red arrows indicate regions of interest.

MRI performed at the time of pain progression did not demonstrate a definite subchondral low-signal band on T1; T2 fat-suppressed (Dixon-water) images showed a heterogeneous marrow signal without focal collapse, albeit limited by motion and constrained hip extension (Figure 5).

### 3.5. Salvage Procedure and Outcome

**Figure 6:**
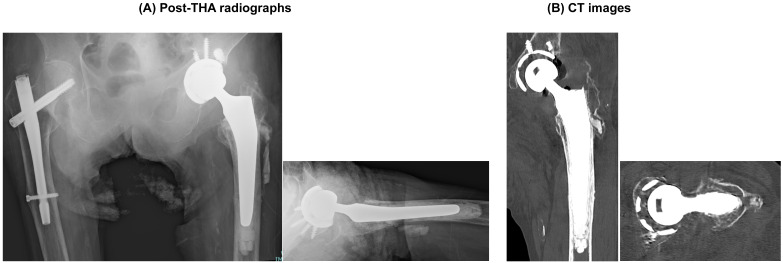
Post-THA radiographs. (**A**) Post-salvage radiographs: AP (**left**) and lateral (**right**) views obtained after conversion to total hip arthroplasty, demonstrating the final component position. (**B**) Representative CT images (coronal reconstruction, **left**; axial slice, **right**).

The patient underwent conversion to total hip arthroplasty (THA), and postoperative AP and lateral radiographs confirmed appropriate component positioning (Figure 6). At the latest follow-up, the pain improved and ambulatory function recovered (indoor ambulation with a walking aid), with stable components and no evidence of migration.

### 3.6. Pathology

**Figure 7:**
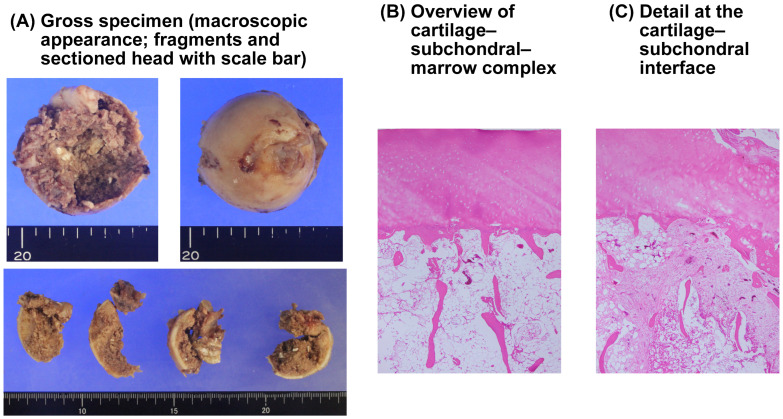
Gross specimen and histopathology of the retrieved femoral head (H&E). (**A**) Gross appearance of the femoral head and representative fragments. (**B**) Overview of the cartilage–subchondral–marrow complex, showing preserved articular cartilage and overall marrow architecture. (**C**) Detail at the cartilage–subchondral interface demonstrating focal reactive fibrovascular change without a confluent necrotic zone and viable osteocytes within lacunae rather than widespread empty lacunae.

Gross inspection of the resected femoral head did not reveal large or well-demarcated necrotic cavities (Figure 7). Decalcified H&E histology across multiple blocks showed preserved osteocytes within many trabeculae, focal reparative fibrovascular changes adjacent to the fracture/implant margin, and no diffuse empty lacunae or frank subchondral collapses. Fibrovascular (granulation) tissue extended in a band-like fashion from the presumed fracture line along the subchondral plate, a pattern most compatible with post-fracture reparative change rather than established primary osteonecrosis, although very early osteonecrotic change cannot be completely excluded. Overall interpretation: No definite avascular necrosis.

## 4. Discussion

The unique failure pattern observed in this case highlights the importance of understanding the potential limitations of cement augmentation for femoral neck preservation. Unlike most previous reports, this case captured a post-union subcapital femoral neck fracture around a cement-augmented helical blade with serial position/alignment metrics and complementary CT, MRI, and histopathological assessments. This observation prompted a deeper investigation into the current literature, as presented in the following review. We conducted a targeted narrative review to examine when malposition negates the apparent protection of cement augmentation in cephalomedullary head fixation and how reduction alignment and telescoping interact with augmented constructs. We extracted clinical and biomechanical reports for the head-element position (Cleveland zones; TAD/CalTAD), reduction (neck–shaft angle), sliding behavior, augmentation use, failure mode, and salvage.

### 4.1. Key Signals from Literature

#### 4.1.1. Importance of Head-Element Positioning

Across multiple series, off-target head element positions (superior and/or anterior on the Cleveland grid), prolonged TAD/CalTAD, and coronal varus reduction have been associated with mechanical failures [10,12,16,17]. Moreover, valgus reduction has been shown to yield a smaller calcar-referenced tip–apex distance and a more inferior femoral neck position of the blade, reinforcing the link between reduction quality and favorable position metrics [18].

In contrast, our index construct began within accepted targets (AP = inferior, lateral = central; TAD 22.6 mm, CalTAD 22.8 mm; NSA 134°) and no overt malposition was present at time zero. However, early sliding was constrained, and progressive varus developed, culminating in neck/subchondral failure. These observations indicate that accurate cephalic-element positioning remains critical and that cement augmentation does not fully mitigate the adverse biomechanics arising from unfavorable positions or altered load transfer.

#### 4.1.2. Preservation of Sliding Reserve and Reduction Quality

Over-valgus reduction or prominent anteromedial cortical support (AMCS) can preload the superior cortex and reduce the available sliding distance (the sliding reserve); therefore, reduction quality substantially influences subsequent sliding and varus behavior [19,20]. With cement augmentation, the head–bone interface is stiffer; therefore, early physiological settling dissipates less via barrel telescoping [13], and the residual bending/shear is expressed as a progressive head–neck varus [15]. When the head element is positioned superior/anterior or excessive CalTAD, peak stresses may shift from the cancellous bed to the adjacent native bone, and the failure mode may change from classic cut-out to femoral neck or subchondral collapse [9,13,15,21].

In our case, true barrel-based telescoping remained ≤ 4 mm for 7 months, while TAD/CalTAD shortened in a pattern compatible with relative proximal migration of the head–cement complex and evolving varus rather than simple controlled impaction. This interpretation is both radiographic and inferential.

#### 4.1.3. What Augmentation Changes—and What It Does Not

Cement augmentation improves rotational stability and reduces micromotion; however, its clinical effects on classic failures are mixed. One RCT suggested fewer mechanical failures with augmentation [7], whereas an RCT-only meta-analysis found no clear reduction in fixation failure or reoperation [22]. Biomechanically, augmentation does not neutralize malposition-induced stress and can shift peak stress to the adjacent native bone, predisposing to neck/subchondral failure when alignment is unfavorable [13,14,15].

In augmented heads, aiming for true central–central is at least as safe—and often preferable—to inferior–central because central–central avoids early abutment that can limit sliding and promote a symmetric cement mantle [15,23]. Regardless of the target choice, a pragmatic exclusion zone remains: avoid superior/anterior placement and keep CalTAD ≤ 25 mm, as long tip–apex distances and superior/anterior positions are linked to failure [10,17]. Equally important is to leave a sliding reserve (do not place the tip abutting the subchondral plate) because stiffer augmented interfaces dissipate less via telescoping [13,15].

In addition to these local mechanical considerations, cement augmentation poses systemic and local risks. Bone cement implantation syndrome is an uncommon but potentially catastrophic perioperative complication of cemented hip arthroplasty, characterized by hypoxemia, hypotension, and cardiovascular instability [24,25]. In addition, cement leakage, thermal injury from the exothermic polymerization of polymethylmethacrylate, embolic events, and stress redistribution to adjacent trabeculae or the subcapital region have been reported and may contribute to non-classic failure patterns, including femoral neck or subchondral fractures.

### 4.2. Mechanistic Considerations and Relation to Prior Reports

**Figure 8:**
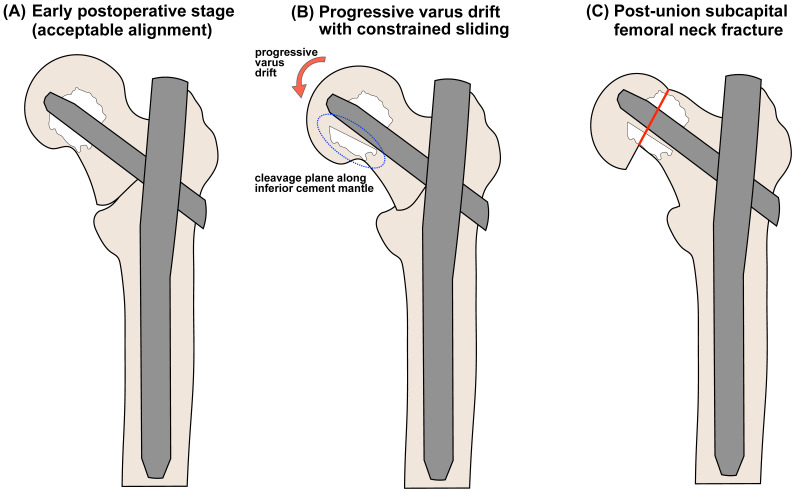
Hypothetical mechanism of post-union subcapital femoral neck fracture around cement-augmented helical blade. (**A**) Early postoperative stage with acceptable alignment and an inferior–central head position. (**B**) Progressive varus drift with constrained sliding and cleavage plane development along the inferior cement mantle. (**C**) Post-union subcapital femoral neck fracture occurring along this plane (red solid line), with the implant retained in situ. This sequence is conceptual and hypothesis-generating.

We acknowledge prior case reports and small clinical series that describe failures around cement-augmented cephalic elements, including cement mantle detachment and blade migration [26,27], most of which occurred before fracture consolidation. Our case differs in that radiographic union of the trochanteric fragment was documented prior to the occurrence of a subsequent subcapital femoral neck fracture, with the cement-augmented helical blade remaining in situ. Rather than asserting the absolute novelty of in situ failure, we frame our contribution as providing serial, metric-based radiographic documentation (TAD/CalTAD/NSA/telescoping measurements across postoperative time points) and a timeline-based hypothesis of a post-union failure pathway. This hypothesized sequence of events is illustrated schematically in Figure 8.

Mechanistically, we propose the following hypothesis-generating interpretation: after apparent trochanteric consolidation, constrained sliding at the head–implant–cement complex (limited telescoping) combined with progressive varus drift may have altered the load transfer such that stresses were concentrated in the subcapital region. Radiographically, this sequence was suggested by limited early barrel-based telescoping (3.8–3.9 mm) with progressive varus and relative proximal migration of the head–cement complex, ultimately followed by a femoral neck fracture in our patient. We emphasize that this interpretation is based on a single clinical observation, remains inferential, and requires biomechanical validation (e.g., cadaveric testing or finite element modeling under constrained sliding) before causal claims are made. Alternative mechanisms, such as micro cut-through of the subchondral bone not resolved on radiographs, focal postoperative osteonecrosis, imperfect cement interdigitation, or measurement/projection error, could also account for the observed failure pattern and cannot be definitively excluded.

### 4.3. Proposed Position- and Sliding-Based Decision Guide (Conceptual)

1. Acceptable position with evidence of early, modest barrel-based sliding on early follow-up radiographs and no load-related pain; WBAT can be continued with routine clinical and radiographic follow-up.
2. Borderline alignment/metrics or early constrained sliding; Examples include a superior/anterior tendency, CalTAD ≥ 25 mm, borderline neck–shaft angle, and limited early barrel-based telescoping. Maintain WBAT; re-verify magnification-corrected TAD/CalTAD; obtain repeat radiographs at 2–3 weeks to assess barrel-based telescoping and neck–shaft angle stability. A low threshold is adopted for early intervention if varus drift or load-related pain occurs.
3. High-risk constellation/red flags; features include load-related pain, progressive varus, lack of demonstrable progression in barrel-based telescoping between the immediate postoperative and 4–6-week radiographs, and/or subchondral stress changes on MRI/CT. Proceed expeditiously with definitive management while maintaining WBAT. In frail osteoporotic patients, THA is often the most reliable option; if refixation is selected, central–central positioning with CalTAD ≤ 25 mm, a symmetric cement mantle, the tip not abutting the subchondral plate, and a sliding-permissive construct are desirable.
4. Index surgery targeting (WBAT-oriented construct design): Prefer true central–central; keep CalTAD ≤ 25 mm; preserve sliding reserve (avoid tip abutment of the subchondral plate). Treat cement augmentation as an adjunct, not a substitute for an accurate trajectory and reduction.

### 4.4. Limitations of the Focused Review

This was a single-patient report coupled with a targeted non-systematic review. Although CT scans were obtained at postoperative week 1 and at 12 and 15 months, serial CT imaging at shorter intervals was not performed because of concerns regarding cumulative radiation exposure. Therefore, we cannot exclude the possibility that subtle internal changes within the cement mantle occurred between the imaging time points. Accordingly, the exact timing of subcapital fractures is uncertain because of their wide imaging intervals. At failure, metal–cement artifacts and fragmentation precluded reliable NSA and TAD/CalTAD measurements. Barrel-based telescoping on radiographs is subject to projection/magnification errors, and early/patchy osteonecrosis or micro-cut-through cannot be entirely excluded. The proposed mechanism is inferential and may not be generalizable to cement-augmented helical blades.

## 5. Conclusions

Cement augmentation can improve cephalic purchase in osteoporotic bones but does not eliminate the need for accurate implant positioning and preservation of the sliding reserve. Based on this single case and the literature reviewed, we propose that aiming for central-central head placement, maintaining a CalTAD ≤ 25 mm, and preserving telescoping may be advisable to reduce the risk of post-union head–cement migration.

## Figures and Tables

**Table 1:** Serial radiographic measurements.

Time Point	TAD (AP + Lat) (mm)	CalTAD (mm)	NSA (°)	Telescoping (mm)	Notes
Postop	22.6	22.8	134	–	Immediate postoperative (Figure 2)
4 months	27.9	25.0	133	3.8	Radiographs (Figure 3A)
7 months	13.8	13.0	131	3.9	Radiographs (Figure 3B)
12 months	N/A	N/A	N/A	N/A *	Radiographic TAD/CalTAD/NSA comparison was precluded by artifact and relative head–cement migration; see * (apparent blade protrusion, 7.4 mm).
15 months (failure)	N/A	N/A	N/A	N/A *	Fragmentation and metal–cement artifact precluded reliable radiographic measurements; see * (apparent blade protrusion, 13.2 mm).

* CT-based apparent blade protrusion at 12 and 15 months (7.4 mm and 13.2 mm, respectively), indicating lateral blade migration due to relative head–cement proximal migration; not directly comparable to radiographic telescoping. Abbreviations: TAD, tip–apex distance; AP, anteroposterior; CalTAD, calcar-referenced tip–apex distance; NSA, neck–shaft angle; N/A, not available.

## Data Availability

Due to ethical considerations, we are unable to make the full dataset publicly available. However, we are open to discussing requests for anonymized data from qualified researchers under appropriate agreements.

## Abbreviations

The following abbreviations are used in this manuscript:

AP	anteroposterior
H&E	hematoxylin and eosin
THA	total hip arthroplasty
IM	intramedullary
TAD	tip–apex distance
CalTAD	calcar-referenced tip–apex distance
NSA	neck–shaft angle
AVN	avascular necrosis
WBAT	weight-bearing as tolerated
PACS	picture archiving and communication system
